# The Sanative Influence of Climate

**Published:** 1846-07

**Authors:** 


ART V.—*The Sanative Influence of Climate.* By Sir JAMES CLARK, Bart., M.D., F.R.S., Physician in Ordinary to the Queen and to the Prince Albert. *Fourth Edition.—London,* 1846. 8vo, pp. 412.

THE almost unexampled event of a genuine medical work—neither a school-book nor a book written expressly for the public—coming to a *fourth edition,* is assuredly a striking indication of its character and the estimation in which it is held, and may well justify our dismissing it with brief notice. In our number for July, 1841 (Vol. XII, p. 160), we gave an outline of the contents of the Third Edition, and an analysis of its principal sections. The present edition is substantially the same work, the former plan being preserved; but the book is much enlarged by the addition of a great deal of new matter of importance, and is moreover much improved by alterations and emendations in every chapter. Among the improvements we may mention new articles on Bournemouth on our south coast, Egypt, and various places in the southern hemisphere; great additions to the article on Madeira; and an entire remodelling of the meteorological tables—now the most extensive and accurate series which have ever been constructed.

Sir James, with characteristic modesty, tells us, in his preface, that "with all the improvements which he has been able to effect in it, the work is still to be regarded only as an essay which future and more extended observations will be required to perfect." We venture to express our belief that it is as complete now as the present state of our knowledge admits of its being made. He adds, that "although he has seen no reason to change his opinions on the characters of the different climates treated of, yet that the information he has continued to receive from others, added to his own increased experience, has enabled him, with more confidence and precision, to lay down rules respecting the adaptation of certain climates to the cure of particular diseases." This is the natural and necessary result of time; and we hope the author may yet find leisure enough to give us yet more, and more complete, editions; but as Sir James has already, we believe, got somewhat beyond the *mezzo del cammin,* and is, moreover, one of the busiest of doctors, we may reasonably enough presume that the work has at length received the author's last touches, and will go down to posterity in its present form—one of the most original and most classical productions of the medical press of our time. It is, indeed, in every way a classical work; and as it is the result of long and accurate observation and experience, and has no connexion with our transitory opinions or professional fashions, it stands a fair chance of being one of the few medical books of the present day which will be familiar to the readers and practitioners of other days. For ourselves, we owe to the work a debt of especial gratitude, as having, in its earliest form of "Notes," full five-and-twenty years ago, directed our attention to the curative influences of Nature in chronic diseases, and thus led us to the adoption of views and plans of treatment, which have been to ourselves the source of infinite gratification, and, through us, may—we would fain hope—be of some slight benefit to the profession and the public. Of the rational system of treating diseases, in accordance with Nature not in opposition to her, the author of this volume has always been the enlightened and consistent advocate; and we believe that for the improvod plan of management in many chronic diseases, now more prevalent in this country than formerly, the profession is not a little indebted to his writings and his example.

We need scarcely add any formal opinion of the volume before us. There are few whom its perusal will not both gratify and instruct. To all who desiderate a knowledge of the sanative influence of climate, both at home and abroad, it is indispensable.

